# Serum Alpha-Klotho and Coronary Atherosclerosis: Associations with Coronary CT Angiography Findings and Atherogenic Indices

**DOI:** 10.3390/biomedicines14092026

**Published:** 2026-09-09

**Authors:** Ayça Tuzcu, Mustafa Gök, Kenan Yörük, Mustafa Yılmaz, Ali Alkaşi, Göksel Tuzcu

**Affiliations:** 1 Department of Biochemistry, Faculty of Medicine, Aydın Adnan Menderes University, 09100 Aydın, Türkiye; atuzcu@adu.edu.tr (A.T.); kenan-1997@hotmail.com (K.Y.); mustafa.yilmaz@adu.edu.tr (M.Y.); 2 Department of Radiology, Faculty of Medicine, Aydın Adnan Menderes University, 09100 Aydın, Türkiye; mustafagok@yahoo.com (M.G.); alialkasi@hotmail.com (A.A.)

**Keywords:** alpha-Klotho, coronary atherosclerosis, coronary artery calcification, Agatston score, CAD-RADS, atherogenic index of plasma, coronary CT angiography

## Abstract

**Background/Objectives**: Alpha-Klotho is a circulating protein with anti-aging and vasculoprotective properties that may play a role in vascular calcification. This study aimed to investigate the association between serum alpha-Klotho levels and coronary atherosclerotic burden assessed by the Agatston score and CAD-RADS grading, as well as cardiometabolic risk indices. **Methods**: This single-center, prospective, cross-sectional study included 86 patients undergoing coronary computed tomography angiography. Serum alpha-Klotho levels were measured by ELISA. Associations with imaging and metabolic variables were assessed using Spearman correlation and age-adjusted partial correlation analyses. Independent associations were evaluated using multivariable linear regression with HC3-robust standard errors. Binary and ordinal logistic regression models with CAD-RADS as the dependent variable and receiver operating characteristic (ROC) analysis were additionally performed. **Results**: Serum Klotho levels were negatively correlated with age (ρ = −0.598), Agatston score (ρ = −0.540), and CAD-RADS (ρ = −0.549) (all *p* < 0.001). Associations with the Agatston score and CAD-RADS remained significant after age adjustment (ρ = −0.348 and −0.352, respectively). AIP also remained independently associated with Klotho after age adjustment (ρ = −0.289, *p* = 0.007), whereas associations with TyG and METS-IR were attenuated. Multivariable models explained 50–52% of the variance in Klotho. In ordinal logistic regression, Klotho remained independently associated with CAD-RADS severity after adjustment for age, sex, BMI, and AIP (OR = 0.525, *p* = 0.033). ROC analysis demonstrated moderate discrimination for the presence of coronary atherosclerosis, defined as Agatston score > 0 (equivalently, CAD-RADS > 0 in this cohort) (AUC = 0.754, 95% CI 0.654–0.855); an exploratory, internally derived cutoff of ≤823 pg/mL—not a clinically validated threshold—yielded 72.0% sensitivity and 75.0% specificity (bootstrap-validated optimism-corrected: 69.3% and 72.5%). **Conclusions**: Lower serum alpha-Klotho levels were independently associated with greater coronary atherosclerotic burden and showed moderate discriminative ability for its presence. These findings are preliminary and support further investigation of alpha-Klotho as a candidate circulating biomarker of coronary atherosclerosis, pending validation in larger, prospective cohorts.

## 1. Introduction

Cardiovascular disease (CVD) remains the leading cause of mortality and morbidity worldwide, making early risk stratification critical for clinical management. Coronary computed tomography angiography (CCTA) has become a gold-standard noninvasive tool for this purpose, yielding two complementary metrics of atherosclerotic burden: the Agatston calcium score, which quantifies calcification on a continuous scale, and CAD-RADS (Coronary Artery Disease Reporting and Data System), an ordinal stenosis grade from 0 (no plaque) to 5 (total occlusion) that standardizes clinical reporting across centers. Both carry strong, independent prognostic value for cardiovascular events [1]. Yet imaging alone describes the anatomical consequence of atherosclerosis, not its biological drivers—understanding the circulating biomarkers that underlie the calcification process could offer pathophysiological insight beyond risk stratification alone.

One such candidate is Klotho, a gene first identified in a mouse model in 1997, whose inactivation produces a phenotype resembling premature aging—shortened lifespan, vascular calcification, osteoporosis, and atherosclerosis [2]. Its membrane-bound form is expressed in a limited set of tissues (renal distal tubules, parathyroid gland, choroid plexus); proteolytic cleavage of the extracellular domain releases a soluble form, s-Klotho, into the circulation, where it acts as an endocrine factor [3]. Beyond serving as a co-receptor for fibroblast growth factor 23 (FGF23) in phosphate-calcium homeostasis, Klotho exerts pleiotropic effects—suppressing oxidative stress, modulating insulin/IGF-1 signaling, and preserving endothelial function [4]. Serum Klotho declines progressively with age, a decline implicated in the pathogenesis of several age-related diseases, including chronic kidney disease, cancer, neurodegenerative disorders, and cardiovascular disease [5].

At the vascular level, Klotho deficiency promotes calcification by driving osteogenic transdifferentiation of vascular smooth muscle cells and impairing autophagy [6], a mechanism corroborated across diverse clinical settings. In patients with stable coronary artery disease, higher serum Klotho levels have tracked with lower intravascular-ultrasound calcification indices [7], while in hemodialysis patients, low Klotho has been linked to greater coronary calcification and worse prognosis [8]. Beyond the coronary bed, reduced circulating Klotho was independently associated with aortoiliac calcification and 10-year mortality in a cohort study [9], and population-based NHANES data have tied low Klotho to established cardiovascular risk factors such as smoking and obesity [10]. Together, these findings converge on a protective role for Klotho in vascular calcification.

The relationship between Klotho and metabolic parameters, by contrast, is considerably less consistent. NHANES data have shown a positive association between HOMA-IR and Klotho in non-diabetic individuals, most pronounced among those who were obese [11], while a separate NHANES analysis of the triglyceride-glucose (TyG) index found the association with Klotho to be negative in non-diabetic participants but positive in those with diabetes—a divergence the authors attributed to compensatory or pathological roles for Klotho at different stages of glucose metabolism [12]. Similarly, a systematic review of obesity and sarcopenic obesity found that, while most studies reported a negative association between s-Klotho and obesity, a minority reported the opposite [13,14]. This heterogeneity suggests that the Klotho-metabolic risk relationship is modified by population characteristics, age, and concomitant metabolic status such as diabetes.

Notably, studies of Klotho and vascular calcification and studies of Klotho and metabolic indices have largely proceeded in parallel rather than in conversation: few cohorts have evaluated CCTA-based imaging findings (Agatston score, CAD-RADS grading) alongside atherogenic/metabolic indices such as the TyG index and the Atherogenic Index of Plasma (AIP) within the same patients. This gap limits a holistic understanding of Klotho’s role across the cardiometabolic risk continuum.

In this study, we investigated the association between serum alpha-Klotho and both the Agatston calcium score and CAD-RADS stenosis grading in a cohort of patients undergoing CCTA, together with its association with cardiometabolic risk indices—the TyG index, AIP, and METS-IR—within the same cohort. We hypothesized that lower Klotho levels would be independently associated with greater coronary atherosclerotic burden and a more adverse cardiometabolic risk profile. Given the well-established influence of age on both Klotho and coronary calcification, a secondary aim was to determine whether these associations persisted after adjustment for age.

This study offers several contributions relative to prior work. First, to our knowledge, this is among the first cohorts to evaluate serum Klotho against both the Agatston calcium score and CAD-RADS stenosis grading within the same CCTA-characterized population, rather than relying on a single imaging modality. Second, we assessed Klotho against multiple cardiometabolic indices (TyG index, AIP, METS-IR) in the same patients, allowing calcification-related and metabolic associations to be evaluated jointly and independently of age within one dataset. Third, beyond modeling Klotho as the outcome of interest, we directly modeled CAD-RADS as the dependent variable (binary and ordinal), complemented by an exploratory ROC-based cutoff and a series of sensitivity analyses—clinically grouped categories, Firth’s penalized regression, and bootstrap validation—that transparently address the constraints of a moderate sample size. Fourth, we identified a significant interaction between diabetes status and the Klotho-TyG association, replicating in an independent, CCTA-characterized cohort a directional pattern previously reported in the general population. Finally, sex emerged as a strong, previously unexamined independent correlate of CAD-RADS severity in this setting, a finding that may inform covariate selection in future Klotho-atherosclerosis studies.

## 2. Materials and Methods

### 2.1. Study Design and Population

This study was designed as a single-center, prospective, cross-sectional observational study. The study protocol was approved by the Non-Interventional Clinical Research Ethics Committee of Aydın Adnan Menderes University Faculty of Medicine (Protocol No: 2026/93, approval date: 2 March 2026). Written informed consent was obtained from all participants, and the study was conducted in accordance with the principles of the Declaration of Helsinki.

Patients who presented for clinically indicated coronary computed tomography angiography (CCTA) during a 3-month enrollment period were consecutively included in the study.

Of the 90 targeted patients, 4 were excluded because the collected blood samples were hemolyzed and therefore could not be reliably analyzed for laboratory parameters, including the Klotho ELISA; the final analysis was completed with *n* = 86 patients.

Inclusion Criteria

- Adults aged 18 years or older;
- Coronary CT angiography clinically indicated or performed;
- Provision of written informed consent after being informed about the study;
- No condition precluding blood sample collection as specified in the study protocol.

Exclusion Criteria

- Age under 18 years;
- Absence of written informed consent;
- Moderate to severe renal impairment (estimated glomerular filtration rate [eGFR] < 60 mL/min/1.73 m^2^) or dialysis dependence;
- History of prior myocardial infarction or coronary revascularization (stenting or bypass surgery);
- Clinically overt heart failure or severe valvular disease;
- Hemodynamic instability;
- Known peripheral artery disease or carotid artery stenosis ≥ 50%;
- Acute infection, active inflammatory disease, or malignancy;
- Current statin therapy (patients receiving statin therapy were excluded to avoid the potential confounding effect of statins on serum lipid parameters and Klotho levels; statins have been shown to upregulate Klotho expression independently of their lipid-lowering effects [15]);
- Systemic diseases likely to significantly affect vascular structure (e.g., advanced liver disease);
- Atrial fibrillation or a significant arrhythmia likely to affect measurements;
- Clinical condition precluding tolerance of the study protocol procedures;
- Incomplete clinical or imaging data.

### 2.2. Sample Size (Power Analysis)

Sample size was calculated a priori based on the effect size (r = −0.315) reported for the association between serum Klotho level and arterial calcification score in a previous study [9]. Using Fisher’s z-transformation with α = 0.05 (two-tailed) and 80% statistical power (1 − β = 0.80), the minimum required sample size was calculated as 77. To account for potential data loss, a target enrollment of 90 patients was set; the study was completed with *n* = 86 patients, exceeding the calculated power threshold.

### 2.3. Coronary Calcium Scoring and CT Angiography Protocol

Prior to contrast-enhanced CCTA, a non-contrast, ECG-gated coronary artery calcium scan was acquired in the same imaging session using prospective (sequential) ECG triggering. Calcium scoring acquisition parameters were as follows: section thickness, 3 mm; tube voltage, 120 kVp; tube current, 250 mA. Coronary artery calcium burden was quantified from these non-contrast images using the standard Agatston method, applying a fixed attenuation threshold of 130 Hounsfield units (HU). All coronary CT angiography examinations were performed using a 160-slice, 128-detector multidetector computed tomography (MDCT) scanner (Aquilion Prime, Toshiba Medical Systems, Otawara, Japan). Iodinated contrast material (iohexol, 350 mg/mL) was administered via the left antecubital vein at a rate of 4 mL/s (70–100 mL total volume) using an automated injection device. Following the onset of contrast infusion, scanning was performed from the cardiac apex to the base using the bolus tracking technique. All examinations were performed in the supine position within a single breath-hold. Scans were acquired with retrospective electrocardiography (ECG)-gated modulation in all patients, with all phases across 0–90% of the R-R interval reconstructed at 10% intervals. As a uniform acquisition protocol was applied across the cohort, gating mode did not represent a potential source of variability in calcium scoring.

Imaging parameters were as follows: section thickness, 0.5 mm; rotation time, 400 ms; 300–400 mAs; 100 kVp; section spacing, 0.25 mm. Axial MDCT sections were transferred to a workstation and evaluated using three-dimensional volume rendering, maximum-intensity projection (MIP), and two-dimensional multiplanar reconstructions (MPR). Vessel segmentation was performed using a dedicated cardiac analysis software (Terarecon-Aquarius Workstation Intuition Edition v.4.4.7.1021.7056).

Coronary artery stenosis was graded according to the standardized CAD-RADS system [16], based on the highest-grade stenosis in each coronary segment, ranging from 0 (no plaque) to 5 (total occlusion) (Table 1). Agatston scoring and CAD-RADS grading were performed by two radiologists (with 20 and 5 years of experience in cardiovascular imaging, respectively). Images were evaluated jointly and scores were assigned by consensus rather than independently; therefore, formal interobserver agreement statistics (e.g., kappa, intraclass correlation coefficient) were not calculated. Because the non-contrast calcium scan was acquired immediately before the contrast-enhanced CCTA within the same imaging session, radiologists were not blinded to the Agatston score when subsequently assigning the CAD-RADS category. CAD-RADS assignment was based on visual assessment of luminal stenosis severity; formal quantitative characterization of non-calcified plaque morphology (e.g., low-attenuation plaque, positive remodeling, napkin-ring sign) was not performed.

For statistical analysis, CAD-RADS 4A and 4B subcategories were combined into a single category (CAD-RADS 4) because of the limited size of these subgroups (*n* = 8 and *n* = 3, respectively). Median Klotho levels were reasonably similar between the two subcategories (4A: 652 pg/mL, IQR 556.5–709.5; 4B: 635 pg/mL, IQR 575.0–721.0), supporting this simplification and indicating that no substantial information was lost by combining them; the combined CAD-RADS 4 category (*n* = 11) had a median of 635 pg/mL (IQR 543.5–718.0).

### 2.4. Laboratory Measurements and Biomarker Calculation

Serum samples were stored at −20 °C for up to one month prior to analysis, in accordance with the manufacturer’s recommendations, and repeated freeze–thaw cycles were avoided. Serum soluble α-Klotho was measured using a sandwich ELISA (Human Soluble α-Klotho/sKLA ELISA Kit, BT LAB Bioassay Technology Laboratory, Shanghai, China, Cat. No. E4142Hu, Lot No. 202601016). The assay has a reported sensitivity of 0.021 ng/mL and a measurement range of 0.05–20 ng/mL. Manufacturer-reported intra-assay CVs range from 2.7% to 5.0%, and the inter-assay CV is <10%. A calibration curve was generated using the kit-provided standards for the analytical run. Samples were analyzed as single measurements without duplicate determination.

Serum triglyceride, HDL-cholesterol, glucose, and CRP levels were measured using standard automated methods on the Abbott Alinity c (clinical chemistry) and Abbott Alinity i (immunoassay) analyzer systems.

We selected the TyG index, AIP, and METS-IR as surrogate markers of insulin resistance and atherogenic dyslipidemia because, unlike HOMA-IR, they do not require a fasting insulin measurement—which was not collected in this cohort—and because all three have been validated as cardiovascular risk indicators in large population-based cohorts, including NHANES.

The following indices were calculated using standard formulas:

- TyG index = Ln[Triglyceride (mg/dL) × Glucose (mg/dL)/2];
- Atherogenic Index of Plasma (AIP) = log_10_(Triglyceride [mmol/L]/HDL [mmol/L]);
- METS-IR = Ln[(2 × Glucose [mg/dL] + Triglyceride [mg/dL]) × BMI]/Ln[HDL (mg/dL)].

Body mass index (BMI) was calculated as weight (kg)/height^2^ (m^2^). Height and weight were recorded at the time of blood sample collection.

### 2.5. Statistical Analysis

The normality of continuous variables was assessed using the Shapiro–Wilk test. Non-normally distributed variables (including Klotho, Agatston score, CAD-RADS, METS-IR, and CRP) are presented as median (interquartile range [IQR]), while normally distributed variables are presented as mean ± standard deviation.

Associations between Klotho and continuous variables (age, Agatston score, TyG, AIP, METS-IR, CRP) were assessed using Spearman’s correlation coefficient. Given the well-established strong influence of age on both Klotho levels and coronary calcification, age-adjusted partial correlations were additionally calculated for the imaging and metabolic/inflammatory variables to assess the influence of age. A Bonferroni correction was applied to control for Type I error arising from multiple comparisons. As an exploratory analysis, effect modification by diabetes-range glucose status on the TyG index-Klotho association was assessed by including a TyG × diabetes-range glucose interaction term in a linear regression model with natural log-transformed Klotho as the dependent variable, using HC3-robust standard errors.

Two-group comparisons (e.g., CAD-RADS = 0 vs. CAD-RADS > 0; Agatston = 0 vs. Agatston > 0) were performed using the Mann–Whitney U test, and differences among more than two categories (CAD-RADS 0–5) were assessed using the Kruskal–Wallis test.

To identify variables independently associated with Klotho, multiple linear regression analyses were constructed with natural log-transformed Klotho level as the dependent variable. Candidate predictors were selected based on clinical relevance (age, given its well-established influence on both Klotho and coronary calcification, and the imaging variable of primary interest) and on their univariate and age-adjusted association with Klotho reported in Section 3.2; AIP was retained given its significant age-adjusted association, whereas TyG, METS-IR, and CRP were not included as model predictors given their attenuation after Bonferroni correction and, in the case of TyG, its high collinearity with AIP. Heteroscedasticity-robust (HC3-type) standard errors were used. Multicollinearity among variables was assessed using the variance inflation factor (VIF); because of the high correlation between the Agatston score and CAD-RADS (ρ = 0.984), these two variables were never included together in the same model and were reported as separate models.

Statistical analyses were performed using Python (version 3.12) with the pandas (version 3.0), SciPy (version 1.17), and statsmodels (version 0.14) libraries; HC3-type heteroscedasticity-robust standard errors were computed using the statsmodels package. A *p*-value < 0.05 was considered statistically significant.

To evaluate whether Klotho level is independently associated with the presence and severity of coronary atherosclerosis—the more directly clinically relevant framing—CAD-RADS was additionally modeled as the dependent variable. A binary logistic regression model (CAD-RADS 0 vs. CAD-RADS > 0) and a multivariable ordinal logistic regression model (proportional-odds model, full CAD-RADS 0–5 scale) were constructed, with natural log-transformed Klotho, age, sex, BMI, and AIP as independent variables. For the logistic regression models, natural log-transformed Klotho was additionally standardized (divided by its standard deviation) so that odds ratios reflect the effect of a 1-SD increase in ln(Klotho). Results are expressed as odds ratios (OR) with 95% confidence intervals. The proportional-odds assumption of the ordinal model was assessed by comparing its fit to an unconstrained multinomial logistic model using a likelihood ratio test. Additionally, receiver operating characteristic (ROC) curve analysis was performed to evaluate the discriminative ability of serum Klotho for the presence of coronary atherosclerosis (Agatston score > 0 and CAD-RADS > 0, which showed complete concordance in the present cohort). The area under the curve (AUC) with 95% confidence interval was calculated, and the optimal cutoff was determined using the Youden index. As the cutoff was derived and evaluated within the same cohort, it should be regarded as an exploratory threshold rather than an externally validated clinical cutoff.

### 2.6. Sensitivity Analyses

Given the moderate sample size and small extreme CAD-RADS subgroups (particularly CAD-RADS 5, *n* = 3), several sensitivity analyses were performed. First, CAD-RADS was regrouped into three clinically meaningful categories (0 = no disease; 1–2 = non-obstructive; 3–5 = obstructive) and the ordinal logistic regression was refitted. Second, Firth’s bias-reduced (penalized) logistic regression [17,18] was applied to the binary CAD-RADS model to address potential small-sample bias. Third, bootstrap resampling (1000 iterations for ROC analysis, 500 for ordinal models) was used to assess the internal stability of the ROC-derived cutoff and of the Klotho odds ratios, and to estimate optimism-corrected sensitivity and specificity.

## 3. Results

### 3.1. Cohort Characteristics

A total of 86 patients were included in the study (50 male [58.1%], 36 female [41.9%]; mean age 59.3 ± 11.4 years). The baseline demographic, laboratory, and imaging characteristics of the cohort are summarized in Table 2.

The median serum alpha-Klotho level was 819 pg/mL (IQR: 703–929). The median Agatston calcium score was 83.5 (IQR: 0–323.5), and 36 patients (41.9%) had an Agatston score of 0 (no calcification). The distribution according to CAD-RADS grading was as follows: CAD-RADS 0, *n* = 36 (41.9%); CAD-RADS 1, *n* = 11 (12.8%); CAD-RADS 2, *n* = 14 (16.3%); CAD-RADS 3, *n* = 11 (12.8%); CAD-RADS 4, *n* = 11 (12.8%; combined 4A and 4B subcategories); CAD-RADS 5, *n* = 3 (3.5%).

### 3.2. Associations Between Klotho and Clinical, Imaging, and Metabolic Parameters

The Shapiro–Wilk test indicated that the majority of variables, including Klotho, Agatston score, CAD-RADS, TyG index, METS-IR, and CRP, were not normally distributed (*p* < 0.05 for all); therefore, Spearman’s correlation coefficient was used, and given the well-established influence of age on both Klotho levels and coronary calcification, age-adjusted partial correlations were additionally calculated for the imaging and metabolic/inflammatory variables (Table 3).

Serum Klotho level showed a strong, negative association with age (ρ = −0.598, *p* < 0.001). Klotho correlated comparably strongly with both imaging variables—the Agatston score (ρ = −0.540, 95% CI −0.674 to −0.370, *p* < 0.001) and CAD-RADS (ρ = −0.549, 95% CI −0.682 to −0.382, *p* < 0.001)—and both associations remained significant with nearly identical effect sizes after age adjustment (ρ = −0.348, 95% CI −0.523 to −0.146, *p* = 0.001 and ρ = −0.352, 95% CI −0.526 to −0.150, *p* < 0.001, respectively), indicating a high degree of concordance between calcification burden and stenosis severity in this cohort. Among the metabolic/atherogenic indices, AIP showed a significant negative association with Klotho both in unadjusted (ρ = −0.435, *p* < 0.001) and age-adjusted analysis (ρ = −0.289, *p* = 0.007). The TyG index was also significantly associated with Klotho in unadjusted analysis (ρ = −0.416, *p* < 0.001), but this weakened to borderline significance after age adjustment (ρ = −0.233, *p* = 0.031). METS-IR showed a weak but significant unadjusted association (ρ = −0.236, *p* = 0.029) that lost significance after age adjustment (ρ = −0.120, *p* = 0.273); no significant association was found with CRP at either stage (ρ = −0.202, *p* = 0.062 unadjusted; ρ = −0.130, *p* = 0.234 age-adjusted) or with BMI (ρ = −0.046, *p* = 0.677).

When a Bonferroni correction was applied across the four metabolic/inflammatory variables (TyG, AIP, METS-IR, CRP; corrected α = 0.0125), only AIP remained significant; the TyG index did not survive this threshold, suggesting that its unadjusted association with Klotho largely reflects age-related confounding. Overall, the direction of all associations was consistently negative: lower serum Klotho levels were associated with older age, greater coronary calcification/stenosis burden, and an unfavorable metabolic profile.

### 3.3. Group Comparisons

Patients with an Agatston score of 0 (*n* = 36, median Klotho = 904 pg/mL) had significantly higher Klotho levels than those with an Agatston score > 0 (*n* = 50, median Klotho = 754 pg/mL) (Mann–Whitney U test, *p* < 0.001). The same comparison repeated for CAD-RADS—CAD-RADS = 0 (*n* = 36, median = 904 pg/mL) versus CAD-RADS > 0 (*n* = 50, median = 754 pg/mL)—yielded an identical result (*p* < 0.001), reflecting the strong concordance between the two scoring methods.

**Figure 1:**
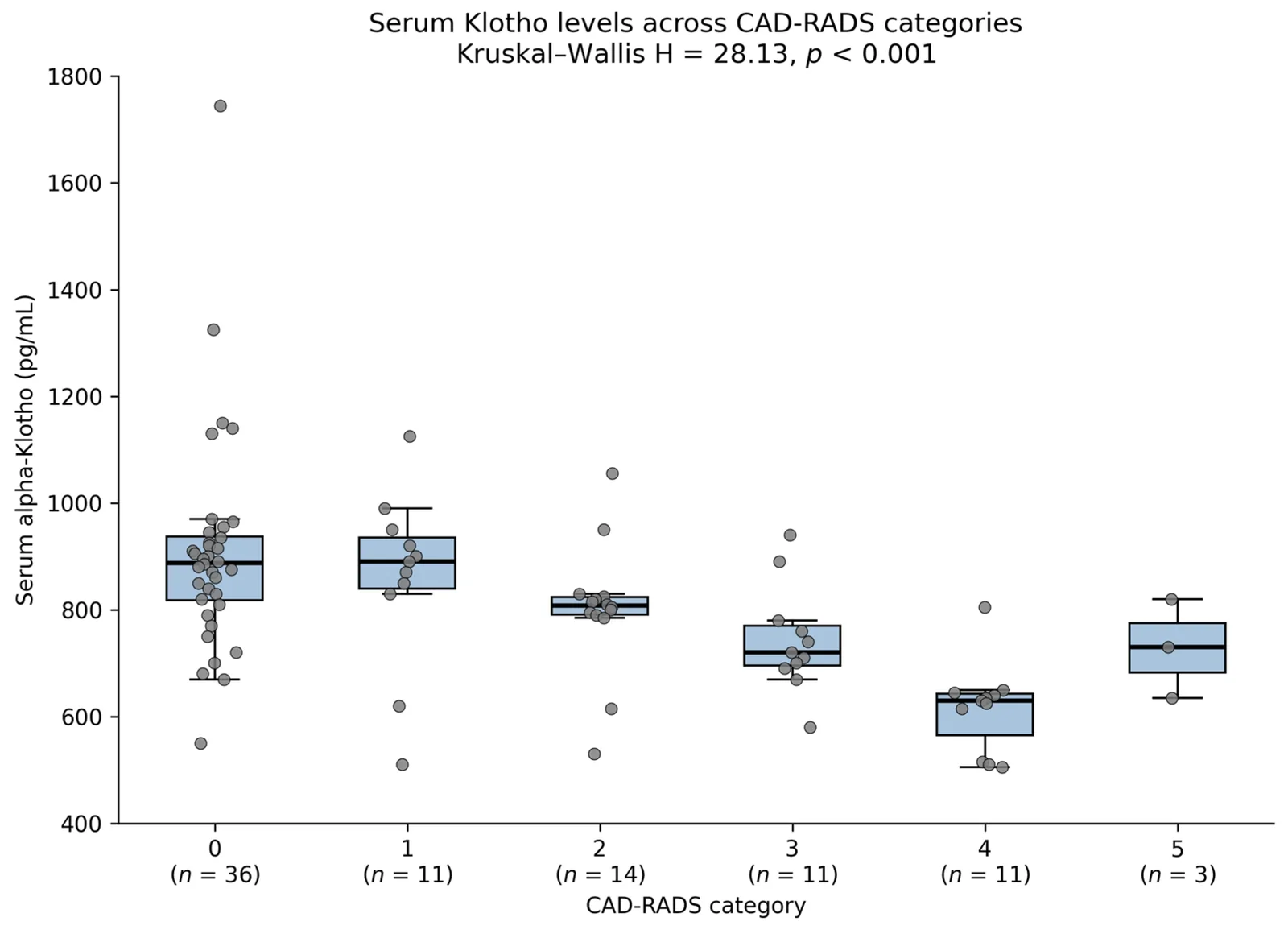
Distribution of serum Klotho levels across CAD-RADS categories (box plot with individual data points). Kruskal–Wallis H = 28.13, *p* < 0.001.

An overall difference in Klotho levels was observed across the six CAD-RADS categories (0–5) (Kruskal–Wallis test, H = 28.13, *p* < 0.001). Median Klotho levels showed a monotonic decreasing trend with increasing CAD-RADS category, from 904 pg/mL in CAD-RADS 0 to 635 pg/mL in CAD-RADS 4; this trend was partially disrupted in the CAD-RADS 5 category (*n* = 3) due to the small subgroup size (median = 733 pg/mL). The distribution of Klotho levels across CAD-RADS categories is shown in Figure 1.

### 3.4. Multivariable Regression Analysis

To identify variables independently associated with natural log-transformed Klotho level, two separate multivariable linear regression models were constructed using HC3-type robust standard errors. Because of the very high correlation between the Agatston score and CAD-RADS (ρ = 0.984), these two variables were never included together in the same model.

Model A (age + log[Agatston + 1] + AIP): The model explained 50.3% of the variance in Klotho (R^2^ = 0.503, adjusted R^2^ = 0.485). Age showed the strongest independent association (standardized β = −0.513, *p* < 0.001), followed by the Agatston score (standardized β= −0.199, *p* = 0.027) and AIP (standardized β = −0.169, *p* = 0.050, borderline significant).

Model B (age + CAD-RADS + AIP): The model explained 51.9% of the variance in Klotho (R^2^ = 0.519, adjusted R^2^ = 0.502)—a slightly higher explanatory power than Model A. Age remained most strongly associated (standardized β = −0.498, *p* < 0.001); CAD-RADS showed a numerically somewhat stronger association with Klotho than the Agatston score did in Model A (standardized β = −0.245, *p* = 0.004); the contribution of AIP was borderline in this model (standardized β = −0.157, *p* = 0.060).

In both models, variance inflation factors (VIF) for all variables remained below 1.5, indicating no clinically meaningful multicollinearity (Table 4).

As sex emerged as strongly and independently associated with CAD-RADS status in the complementary analyses described in Section 3.5, a sensitivity analysis was performed to assess whether sex confounded or modified the associations reported in Models A and B. Serum Klotho levels did not differ significantly between men and women in unadjusted comparison (median 792 vs. 870 pg/mL; Mann–Whitney *p* = 0.060). When sex was added as an additional covariate, it was not independently associated with natural log-transformed Klotho in either model (Model A: *p* = 0.156; Model B: *p* = 0.317). The association between Klotho and the Agatston score was slightly attenuated (standardized β = −0.160, *p* = 0.059) and remained essentially unchanged for age (*p* < 0.001); the association with CAD-RADS remained statistically significant (standardized β = −0.204, *p* = 0.038), and model fit was not materially improved (R^2^ change ≤ 0.013 in both models). These findings indicate that sex is independently associated with the presence of coronary atherosclerosis itself, but not independently associated with circulating Klotho levels once age, imaging burden, and AIP are accounted for.

Diabetes-range fasting glucose (≥126 mg/dL) was present in 13 patients (15.1%). Serum Klotho levels did not differ significantly between patients with and without diabetes-range glucose (median 823 vs. 807 pg/mL; Mann–Whitney *p* = 0.852), and this status was not independently associated with Klotho when added to Models A and B (*p* = 0.330 and *p* = 0.404, respectively). However, the association between the TyG index and Klotho differed significantly according to glucose status (interaction β = 0.275, HC3 SE = 0.099, *p* = 0.006): among patients without diabetes-range glucose, Klotho correlated negatively with the TyG index (ρ = −0.503, *p* < 0.001), whereas this association reversed in direction among the small subgroup with diabetes-range glucose (ρ = 0.269, *p* = 0.374; *n* = 13). Given the limited size of this subgroup (*n* = 13), this finding should be regarded as exploratory and hypothesis-generating rather than confirmatory, and the sample size available for this comparison likely limits statistical power to reliably detect interaction effects. This pattern is consistent with previous reports of diabetes-status-dependent, divergent associations between the TyG index and Klotho.

**Figure 2:**
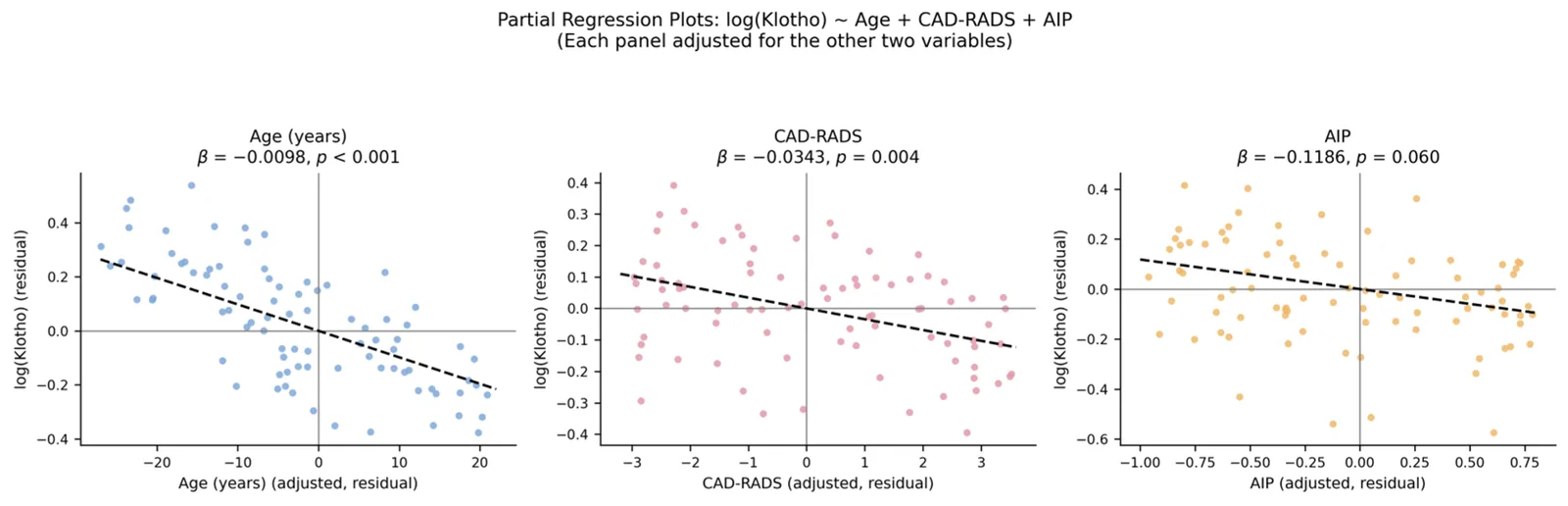
Partial regression plots for Model B: log(Klotho) ~ age + CAD-RADS + AIP. Each panel shows the association after adjusting for the other two variables. The dashed line represents the fitted linear regression (partial regression) line for each relationship. Point color denotes the panel’s predictor variable only (blue = Age, pink = CAD-RADS, orange = AIP) and does not indicate patient subgroups; all 86 patients are plotted in each panel.

The partial regression plots for Model B visually confirm the independent association of each variable (age, CAD-RADS, AIP) with log(Klotho) after adjustment for the other two (Figure 2).

### 3.5. Independent Association of Klotho with Coronary Atherosclerosis

In the analyses presented above, Klotho was modeled as the dependent variable. To address the more directly clinically relevant question—whether Klotho is independently associated with the presence and severity of coronary atherosclerosis—complementary models were constructed with CAD-RADS as the dependent variable.

In the binary logistic regression model (CAD-RADS 0 vs. >0), age (OR = 1.14, 95% CI 1.06–1.23, *p* = 0.001) and sex (male; OR = 8.10, 95% CI 2.25–29.2, *p* = 0.001) were significantly and independently associated with CAD-RADS status, whereas the contribution of Klotho did not reach statistical significance in this model (OR = 0.590, 95% CI 0.256–1.357, *p* = 0.214).

In contrast, in the ordinal logistic regression model that preserves the full categorical information of CAD-RADS (0–5), Klotho remained significantly and independently associated with CAD-RADS after adjustment for age, sex, BMI, and AIP (OR = 0.525, 95% CI 0.290–0.949, *p* = 0.033). Age (OR = 1.10, *p* = 0.001) and sex (OR = 4.97, *p* = 0.001) were consistently and strongly associated with CAD-RADS in both models, while BMI and AIP did not reach significance in either model.

This finding indicates that the association between Klotho and coronary atherosclerotic severity is sensitive to how the outcome is coded: it remains significant when the full ordinal scale is used but loses significance when CAD-RADS is dichotomized, possibly reflecting reduced statistical power from collapsing categorical information. Furthermore, this analysis identified sex—not evaluated in the earlier models—as strongly and independently associated with coronary atherosclerosis (Table 5).

### 3.6. Discriminative Ability of Klotho for Coronary Atherosclerosis Presence

In this cohort, the presence of coronary calcification (Agatston score > 0) and the presence of any coronary stenosis (CAD-RADS > 0) identified the same 50 patients (100% concordance), allowing a single receiver operating characteristic (ROC) analysis to be performed for the presence of coronary atherosclerosis.

**Figure 3:**
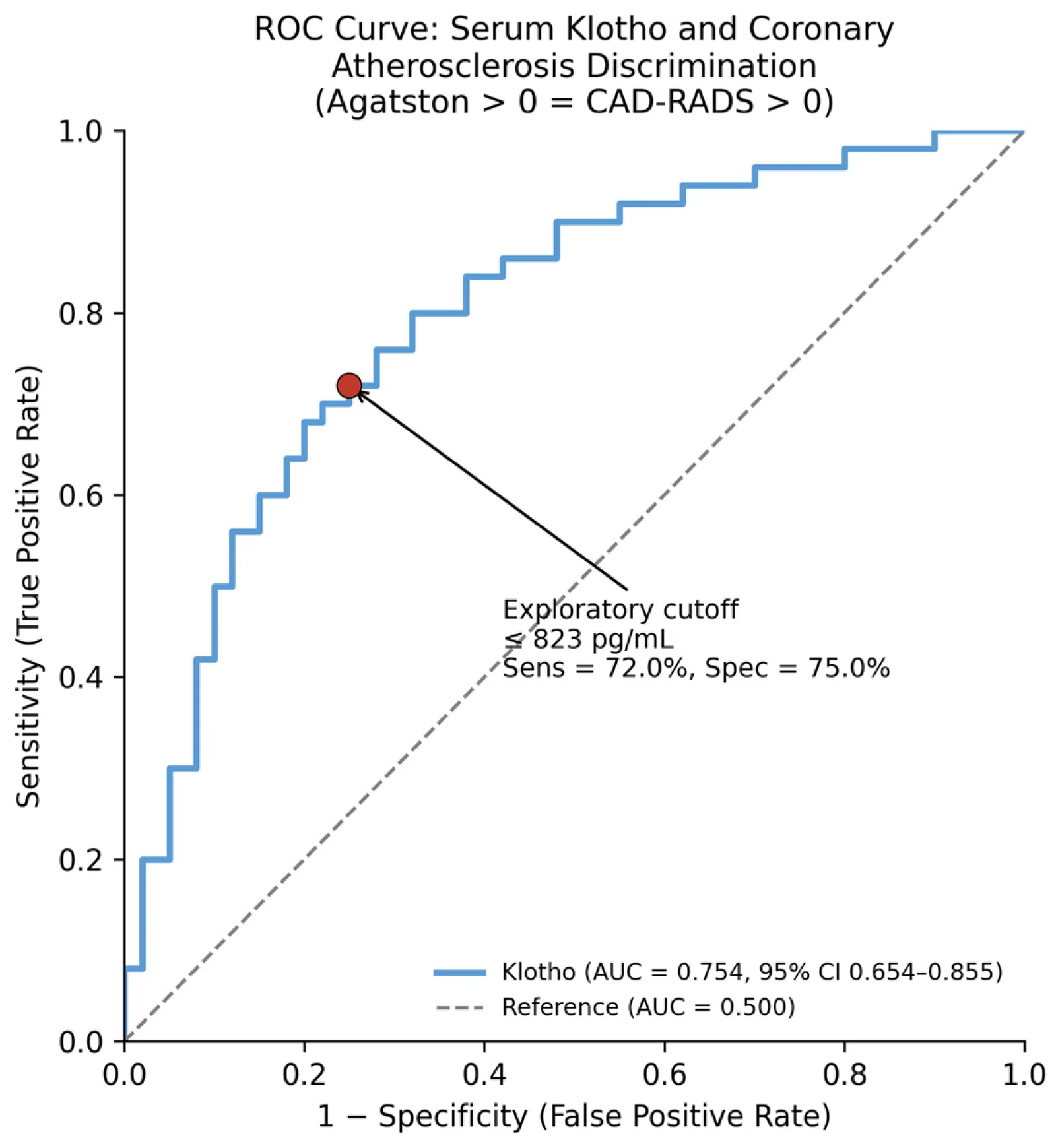
ROC curve for serum Klotho discriminating the presence of coronary atherosclerosis (Agatston score > 0 = CAD-RADS > 0). AUC = 0.754 (95% CI 0.654–0.855). The red dot indicates the Youden-index-derived optimal (exploratory) cutoff point (≤823 pg/mL); arrows connect this point to the corresponding sensitivity and specificity values shown on the plot.

Serum Klotho showed acceptable discriminative ability for the presence of coronary atherosclerosis, with an area under the curve (AUC) of 0.754 (95% CI 0.654–0.855, *p* < 0.001) (Figure 3). The exploratory cutoff value, determined by the Youden index within this cohort, was ≤823 pg/mL, and overall accuracy was 73.3%. This cutoff was subjected to internal bootstrap validation (Section 3.7); however, in the absence of external validation in an independent cohort, it should not be used as a clinical decision point at this stage.

### 3.7. Sensitivity Analyses

Regrouping CAD-RADS into three clinically meaningful categories (0/1–2/3–5) yielded a Klotho odds ratio of 0.491 (95% CI 0.258–0.936, *p* = 0.031), closely replicating the result of the full 6-category ordinal model (OR = 0.525, *p* = 0.033) and confirming that the proportional-odds assumption remained supported (likelihood ratio test *p* = 0.273). Firth’s penalized logistic regression [17,18] applied to the binary model shrank the sex odds ratio from 8.10 (95% CI 2.25–29.2) to 6.70 (95% CI 1.98–22.7), consistent with correction of small-sample bias, while the (non-significant) Klotho estimate was materially unchanged (OR = 0.624 vs. 0.590). Bootstrap resampling (1000 iterations) of the ROC-derived cutoff yielded a highly stable median cutoff of 823 ng/mL (interquartile range 820–823), with modest optimism-corrected sensitivity and specificity of 69.3% and 72.5%, respectively (apparent values: 72.0% and 75.0%). Bootstrap stability analysis of the ordinal Klotho odds ratio confirmed a consistent direction of association across resamples (median OR 0.45–0.49 in both the grouped and full models) but showed that statistical significance (*p* < 0.05) was achieved in only 63% of bootstrap samples, indicating that while the direction and magnitude of the association are robust, its statistical significance should be interpreted with appropriate caution given the sample size.

## 4. Discussion

In this cohort of 86 patients undergoing coronary CT angiography, serum alpha-Klotho level was negatively associated with age, coronary atherosclerotic burden as assessed by the Agatston score (originally described for quantifying coronary calcium using ultrafast CT [19]) and CAD-RADS grading, and the Atherogenic Index of Plasma (AIP). Associations with both imaging measures remained significant after age adjustment and in multivariable analyses, whereas the contribution of AIP—though significant in age-adjusted partial correlation (*p* = 0.007)—was more modest and borderline in the multivariable models (*p* = 0.050–0.060). Together, these variables explained approximately 50–52% of the variance in Klotho in multivariable regression models.

An important methodological finding of this study is that the Agatston score and CAD-RADS grading showed an almost identical pattern of association within this cohort (unadjusted ρ = −0.540 vs. −0.549; age-adjusted ρ = −0.348 vs. −0.352). This indicates a high degree of concordance between calcification burden and stenosis severity in this cohort, although the two metrics are not methodologically interchangeable—Agatston scoring quantifies calcified plaque alone, whereas CAD-RADS reflects overall stenosis severity including non-calcified plaque. Notably, CAD-RADS showed a numerically somewhat stronger association with Klotho than the Agatston score in the respective models (standardized β = −0.245 vs. −0.199; R^2^ = 0.519 vs. 0.503), although given the near-collinearity between the two measures (ρ = 0.984), this difference should not be over-interpreted as evidence of a meaningfully stronger association.

The negative direction of association we observed—lower Klotho levels associated with greater coronary calcification/stenosis burden—is consistent with the dominant body of literature indicating that Klotho plays a protective role in vascular aging and calcification. In a study using intravascular ultrasound, patients with stable coronary artery disease and high serum Klotho levels had significantly lower calcification indices [7]. Low Klotho levels have been associated with increased coronary artery calcification in hemodialysis patients [8], and a recent cohort study demonstrated that reduced circulating Klotho levels were independently associated with aortoiliac calcification and 10-year mortality [9]. In a study directly relevant to our findings, Navarro-González et al. reported that low Klotho levels were associated with both the presence and severity of coronary artery disease [20]—a precedent that directly supports the dose–response relationship we observed with CAD-RADS grading. Furthermore, a recent meta-analysis encompassing 62 studies and more than 27,000 participants reported a significant pooled negative correlation between Klotho and arterial calcification (r = −0.388) [21], indicating that our findings are part of a broad evidence base rather than an isolated observation.

The relationship between Klotho and metabolic parameters presents a more mixed picture in the literature. Some NHANES-based studies have reported a positive association between Klotho and insulin resistance, particularly pronounced in the obese, non-diabetic subgroup [11], while another NHANES analysis examining the TyG index found associations that varied in direction depending on diabetes status [12]. Given this heterogeneity, the metabolic associations reported here should be regarded as hypothesis-generating rather than definitive, and the mechanistic interpretations offered below are intended to motivate future investigation rather than to establish causal pathways. In our cohort, associations with TyG and METS-IR were significant in unadjusted analyses but weakened or disappeared after age adjustment. In contrast, the consistent, age-independent negative association we found with AIP is in line with a large NHANES-based study showing that Klotho is inversely related to triglyceride levels and overall atherogenic lipid profile; in that study, a doubling of serum Klotho concentration was associated with significantly lower triglyceride levels [22]. Given that AIP is derived from the triglyceride-to-HDL ratio, this finding is mechanistically consistent with our results. A plausible explanation for this divergence lies in the compositional difference between the two indices: TyG incorporates fasting glucose, whereas AIP is derived purely from lipid parameters. Because glucose homeostasis itself declines markedly with age—through progressive insulin resistance and reduced pancreatic β-cell function—much of the raw association between TyG and Klotho may be statistically entangled with age rather than reflecting an independent metabolic pathway. This is further complicated by Klotho’s established role in insulin/IGF-1 signaling, raising the possibility that part of the age-adjustment procedure removes not only confounding but also a genuine, age-mediated component of the Klotho-glucose relationship. In contrast, AIP reflects atherogenic dyslipidemia—driven by hepatic triglyceride-rich lipoprotein production and HDL catabolism—a lipid-handling axis that is mechanistically closer to vascular biology and less directly entangled with age-related glycemic decline, which may explain its more robust, age-independent association with Klotho. Our own data provide direct support for this pattern: the association between the TyG index and Klotho differed significantly by diabetes-range glucose status (interaction *p* = 0.006), being negative among patients without diabetes-range fasting glucose but reversing in direction among the small subgroup with diabetes-range fasting glucose (*n* = 13), mirroring the diabetes-dependent divergence reported by Qiu et al. [12], although the small size of our diabetes-range subgroup (*n* = 13) precludes strong biological inference and this observation warrants confirmation in larger samples. Additionally, the exclusion of patients on statin therapy strengthens the internal validity of both the AIP and TyG-related associations reported here, given that statins are a major modifier of lipid profiles. Although the exclusion of patients receiving statins reduced pharmacologic confounding of lipid-derived indices, it may also have introduced a degree of selection bias, limiting the generalizability of our findings to an unselected CCTA population in which statin use is common.

A systematic review on obesity and sarcopenic obesity reported that the majority of included studies found a negative association between Klotho and obesity [13], although some studies reported findings in the opposite direction [14]. The absence of a significant association between BMI and Klotho in our cohort (ρ = −0.046, *p* = 0.677) adds a further data point to this mixed literature.

The Agatston score has long served as the gold standard for cardiovascular risk stratification, with its association with clinical outcomes well established in large cohort studies [1]. The current conceptual framework and clinical applications of coronary artery calcification have been comprehensively reviewed elsewhere [23]. CAD-RADS is a more recent standardized reporting system that more directly links stenosis severity to clinical decision-making [16]. The near-complete overlap in the association pattern between the two methods and Klotho in our study (multicollinearity ρ = 0.984) indicates a high degree of concordance between calcification burden and stenosis severity in this cohort, rather than methodological equivalence between the two metrics. A plausible biological explanation for the marginally stronger performance of CAD-RADS is that it captures luminal stenosis arising from the full spectrum of plaque composition—calcified, non-calcified, and mixed—whereas the Agatston score quantifies calcified plaque burden alone. If Klotho’s vasculoprotective effects extend beyond inhibiting calcification per se to broader anti-atherogenic actions on the vessel wall (e.g., endothelial preservation, smooth muscle phenotype maintenance), a stenosis-based metric may more completely capture the biological process that Klotho influences.

The primary analyses presented above modeled Klotho as the dependent variable; however, the more directly clinically relevant question is whether Klotho is independently associated with the presence and severity of coronary atherosclerosis. Complementary analyses modeling CAD-RADS as the dependent variable showed that this association is sensitive to outcome coding: Klotho remained independently associated with CAD-RADS, adjusted for age, sex, BMI, and AIP, in the ordinal logistic regression model using the full CAD-RADS scale (*p* = 0.033), but lost statistical significance in the binary logistic model (CAD-RADS 0 vs. >0; *p* = 0.214), possibly reflecting reduced statistical power from dichotomizing an ordinal outcome. An alternative, biologically plausible interpretation is that Klotho is more closely tied to the severity or burden of coronary atherosclerosis than to its mere presence or absence—consistent with a graded, dose-dependent relationship rather than a simple threshold effect. This finding supports the robustness of the Klotho-atherosclerosis association at the full-scale level while indicating that confirmation in larger samples using binary outcome definitions is warranted.

This analysis also identified sex as strongly and independently associated with coronary atherosclerosis (ordinal model OR = 4.97, *p* = 0.001), a variable not evaluated in the earlier Klotho-dependent models. This finding is consistent with well-established sex-based differences in cardiovascular risk and suggests that future Klotho-atherosclerosis studies should routinely include sex as a covariate. Notably, a sensitivity analysis showed that sex was not independently associated with Klotho levels themselves (*p* = 0.156–0.317 across models), indicating that its influence operates primarily through its association with atherosclerotic burden rather than through a direct effect on circulating Klotho. This pattern is biologically plausible: established sex differences in atherosclerosis risk are largely attributed to sex-hormone-mediated pathways—such as the vasoprotective effects of estrogen in premenopausal physiology and differences in visceral adiposity and lipid handling—that act on the vessel wall independently of the Klotho axis. These findings suggest that sex-related differences in atherosclerotic burden may not be primarily mediated through circulating Klotho, although the present study was not designed to formally assess mediation.

Beyond formal regression modeling, receiver operating characteristic curve analysis demonstrated that serum Klotho has acceptable discriminative ability for the presence of coronary atherosclerosis (AUC = 0.754, 95% CI 0.654–0.855), with a cutoff of ≤823 pg/mL yielding a sensitivity of 72.0% and specificity of 75.0%. While these operating characteristics are not yet sufficient to support Klotho as a standalone diagnostic test, they provide an exploratory threshold for evaluation in independent cohorts.

Beyond calcification and stenosis severity, CCTA can provide additional biological information relevant to atherosclerotic risk, such as pericoronary fat attenuation index (FAI), a marker of local vascular inflammation [24]. This study did not incorporate FAI or other inflammation-based CCTA metrics, and future work integrating Klotho with such imaging biomarkers may offer a more complete picture of the interplay between systemic biomarkers and local vascular biology.

It is important to situate the present findings within the broader landscape of Klotho research. Several of the studies most directly relevant to our results were substantially larger or drawn from different clinical populations—for example, the meta-analysis by Wungu et al. encompassed 62 studies and more than 27,000 participants [21], and studies in hemodialysis populations have examined Klotho in patients with far more advanced and homogeneous vascular calcification than our CCTA-referred cohort [8]. Population-based cohorts such as Hellou et al. further benefited from substantially larger sample sizes and longitudinal, outcome-based endpoints (mortality) [9]. By contrast, the present study offers a smaller, single-center, imaging-based cross-sectional snapshot in a population undergoing clinically indicated CCTA. We view our findings as complementary to, rather than a replacement for, this larger evidence base: they extend it to a CCTA-characterized population combining calcification and stenosis phenotyping with cardiometabolic indices, but they remain preliminary and, unlike the population-based cohorts cited above, have not yet been linked to hard clinical outcomes.

A key strength of this study is the consistency of direction: all associations between Klotho and the examined variables were consistently negative, aligning with the dominant direction reported in the literature—particularly the CAD severity finding of Navarro-González et al. [20] and the meta-analytic synthesis of Wungu et al. [21]. The persistence of significant associations between Klotho and both CAD-RADS and the Agatston score after age adjustment indicates that these findings cannot be explained by age-related confounding alone. The multivariable model’s ability to explain more than half of the variance in Klotho (R^2^ > 0.50), despite a moderate sample size (*n* = 86), is a notable finding.

The most important limitation of this study is its cross-sectional design, which precludes establishing the direction of causality between Klotho levels and coronary calcification/stenosis or metabolic parameters. Indeed, a Mendelian randomization study investigating the potential causal relationship between circulating Klotho levels and cardiovascular disease has demonstrated the value of evidence types that move beyond cross-sectional observational designs in this field [25]; incorporating similar approaches in future studies could help strengthen the causal interpretation of our findings.

Although the sample size (*n* = 86) exceeded the a priori power calculation, it remains moderate, and the limited size of certain subcategories—particularly CAD-RADS 5 (*n* = 3) complicates the interpretation of results in these groups. Because of the potentially high correlation between the TyG index and AIP, these two variables were not included together in the same regression model. Similarly, the very high correlation between the Agatston score and CAD-RADS (ρ = 0.984) means that the independent prognostic contribution of these two imaging variables cannot be disentangled within this study design. The proportional-odds assumption of the ordinal logistic model was supported by a likelihood ratio test comparing it with an unconstrained multinomial model (*p* = 0.222), although coefficient stability could not be reliably assessed at the extreme CAD-RADS 5 threshold given its very small subgroup size (*n* = 3). Because CAD-RADS and Agatston scores were assigned by consensus reading rather than independently by each radiologist, formal interobserver agreement could not be quantified. The two readers had differing levels of experience in cardiovascular imaging (20 and 5 years); although consensus reading with a senior radiologist likely minimized scoring variability, this cannot be formally confirmed in the absence of independent reads. Furthermore, because the non-contrast calcium scan was performed immediately before the contrast-enhanced CCTA within the same imaging session, readers were not blinded to the Agatston score when assigning the CAD-RADS category, which may have introduced detection bias favoring concordance between the two measures. Formal quantitative characterization of non-calcified plaque (e.g., low-attenuation plaque, positive remodeling) was not performed, as CAD-RADS assignment relied on visual stenosis grading alone. Obstructive, inflamed non-calcified plaques have been reported in patients with a calcium score of zero [26], and although review of the original radiology reports confirmed no non-calcified plaque was documented in any of the 36 patients with Agatston score = 0, the possibility of under-recognized non-calcified disease cannot be entirely excluded given the absence of blinded, independent reads and dedicated plaque-characterization protocols in this study. To address concerns regarding statistical power for regression and ROC analyses, we performed grouped-category, penalized (Firth), and bootstrap sensitivity analyses (Section 3.7); these confirmed the direction and approximate magnitude of the Klotho-CAD-RADS association but also revealed that its statistical significance was not consistently reproduced across bootstrap resamples (63% of 500 resamples), underscoring the exploratory nature of this finding pending validation in a larger cohort.

Serum Klotho levels were determined using a single ELISA measurement without replicate testing; given manufacturer-reported intra-assay CVs of 2.7–5.0%, this may have introduced additional measurement noise into the reported correlations and regression estimates, potentially attenuating the observed associations. Additionally, the exclusion of all patients receiving statin therapy, while strengthening the internal validity of lipid-derived associations, limits the generalizability of our findings to routine CCTA populations, in which statin use is common. The study was conducted at a single center, which may limit the generalizability of the findings. Finally, potential confounding factors that may affect Klotho levels—such as smoking status, hypertension, LDL-cholesterol, medication use, and renal function (e.g., estimated glomerular filtration rate); mineral metabolism parameters relevant to Klotho physiology, including phosphate, vitamin D, PTH, and FGF23, were similarly not systematically available and could not be included as covariates. Additionally, unmeasured lifestyle and constitutional factors—including diet, physical activity level, family history of cardiovascular disease, and subclinical inflammation—were not assessed and likely represent residual confounding that could influence both Klotho levels and coronary calcification, further limiting causal inference beyond the cross-sectional design itself. Similarly, only 13 patients (15.1%) had diabetes-range fasting glucose, limiting the statistical power available to characterize the diabetes-dependent divergence observed in the TyG index-Klotho relationship.

## 5. Conclusions

This study demonstrates that serum alpha-Klotho level is consistently and negatively associated, independent of age, with coronary atherosclerotic burden as measured by both the Agatston score and CAD-RADS grading, and shows moderate discriminative ability for its presence (AUC = 0.754, exploratory cutoff ≤ 823 ng/mL). Beyond this principal finding, two observations extend current understanding of Klotho’s cardiometabolic role: sex emerged as a strong, previously unexamined independent correlate of CAD-RADS severity, and the Klotho-TyG index association diverged by diabetes status, replicating a pattern previously reported only in the general population. Sensitivity analyses (grouped-category, penalized, and bootstrap modeling) confirmed the direction and approximate magnitude of the principal association while transparently characterizing its statistical stability at this sample size. Together, these findings position circulating alpha-Klotho as an imaging-correlated biomarker of coronary atherosclerotic burden and identify sex and diabetes status as important covariates for future Klotho-atherosclerosis research. These findings are preliminary. Larger, multicenter, prospective outcome studies—ideally incorporating causal inference approaches such as Mendelian randomization—are needed to confirm the association between alpha-Klotho and coronary atherosclerosis, and demonstration of incremental predictive value over established cardiovascular risk scores will be a necessary further step before any consideration of clinical implementation.

## Figures and Tables

**Table 1:** CAD-RADS classification categories.

Score	Stenosis	Interpretation
0	0%	Absence of CAD
1	1–24%	Minimal non-obstructive CAD
2	25–49%	Mild non-obstructive CAD
3	50–69%	Moderate stenosis
4A	70–99% (single or 2-vessel)	Severe stenosis
4B	Left main > 50% or 3-vessel ≥ 70%	Severe stenosis
5	100%	Total coronary occlusion

**Table 2:** Baseline characteristics of the cohort.

Variable	Value
Age (years), mean ± SD	59.3 ± 11.4
Sex, Male, *n* (%)	50 (58.1)
Sex, Female, *n* (%)	36 (41.9)
Klotho (pg/mL), median (IQR)	819 (703–929)
Agatston score, median (IQR)	83.5 (0–323.5)
Agatston = 0, *n* (%)	36 (41.9)
CAD-RADS 0, *n* (%)	36 (41.9)
CAD-RADS 1, *n* (%)	11 (12.8)
CAD-RADS 2, *n* (%)	14 (16.3)
CAD-RADS 3, *n* (%)	11 (12.8)
CAD-RADS 4, *n* (%)	11 (12.8)
CAD-RADS 5, *n* (%)	3 (3.5)
Triglyceride (mg/dL), median (IQR)	189 (117.3–285.8)
HDL (mg/dL), median (IQR)	40 (38.0–49.8)
Glucose (mg/dL), median (IQR)	93 (83.3–108.8)
TyG index, median (IQR)	9.24 (8.72–9.49)
AIP, mean ± SD	0.26 ± 0.30
CRP (mg/dL), median (IQR)	0.17 (0.10–0.40)
BMI (kg/m^2^), median (IQR)	28.7 (26.0–31.5)
METS-IR, median (IQR)	44.4 (41.1–53.8)
Diabetes-range fasting glucose (≥126 mg/dL), *n* (%)	13 (15.1)

**Table 3:** Spearman correlations between Klotho and study variables (unadjusted and age-adjusted).

Variable	Unadj. ρ	(95% CI)	Unadj. *p*	Age-Adj. ρ	(95% CI)	Age-Adj. *p*
Age	−0.598	(−0.719, −0.442)	<0.001	–	–	–
Agatston score	−0.540	(−0.674, −0.370)	<0.001	−0.348	(−0.523, −0.146)	0.001
CAD-RADS	−0.549	(−0.682, −0.382)	<0.001	−0.352	(−0.526, −0.150)	<0.001
TyG index	−0.416	(−0.577, −0.224)	<0.001	−0.233	(−0.425, −0.021)	0.031
AIP	−0.435	(−0.592, −0.246)	<0.001	−0.289	(−0.473, −0.081)	0.007 *
METS-IR	−0.236	(−0.426, −0.025)	0.029	−0.120	(−0.324, 0.096)	0.273
CRP	−0.202	(−0.397, 0.010)	0.062	−0.130	(−0.334, 0.086)	0.234
BMI	−0.046	(−0.255, 0.168)	0.677	–	–	–

IQR: interquartile range; ρ: Spearman correlation coefficient; 95% CI: 95% confidence interval (Fisher z-transformation). Age-adjusted values are partial Spearman correlations controlling for age; not computed for Age and BMI (–). * Significant below the Bonferroni-corrected threshold (α = 0.0125) for 4 metabolic/inflammatory variables.

**Table 4:** Multivariable regression models (dependent variable: log[Klotho]).

Model	Variable	Standardized β (95% CI)	*p*
A (R^2^ = 0.503)	Age	−0.513 (−0.754 to −0.271)	<0.001
	log(Agatston + 1)	−0.199 (−0.376 to −0.023)	0.027
	AIP	−0.169 (−0.338 to 0.000)	0.050
B (R^2^ = 0.519)	Age	−0.498 (−0.735 to −0.262)	<0.001
	CAD-RADS	−0.245 (−0.413 to −0.076)	0.004
	AIP	−0.157 (−0.321 to 0.006)	0.060

Model A: age + log(Agatston + 1) + AIP; Model B: age + CAD-RADS + AIP. HC3-type robust standard errors were used. VIF < 1.5 in both models.

**Table 5:** Binary and ordinal logistic regression models for CAD-RADS.

Variable	Binary OR	(95% CI)	*p*	Ordinal OR	(95% CI)	*p*
ln(Klotho), per 1-SD	0.590	(0.256, 1.357)	0.214	0.525	(0.290, 0.949)	0.033
Age, per 1-SD	4.498	(1.892, 10.695)	<0.001	2.807	(1.518, 5.192)	0.001
Sex (male)	8.103	(2.247, 29.215)	0.001	4.972	(1.941, 12.735)	<0.001
BMI, per 1-SD	0.887	(0.516, 1.525)	0.664	1.027	(0.667, 1.581)	0.903
AIP, per 1-SD	0.697	(0.370, 1.312)	0.264	0.940	(0.601, 1.472)	0.788

Binary model: CAD-RADS = 0 vs. CAD-RADS > 0 (logistic regression). Ordinal model: full CAD-RADS scale (0–5; ordinal logistic regression). All continuous predictors (Klotho, age, BMI, AIP) are standardized (per 1-SD); sex is a categorical covariate (male vs. female) and requires no standardization. This unit convention is consistent with Table 4.

## Data Availability

The data that support the findings of this study are available from the corresponding author upon reasonable request.

## Abbreviations

The following abbreviations are used in this manuscript:

AIP	Atherogenic Index of Plasma
AUC	Area Under the Curve
BMI	Body Mass Index
CAD-RADS	Coronary Artery Disease Reporting and Data System
CCTA	Coronary Computed Tomography Angiography
CI	Confidence Interval
CRP	C-Reactive Protein
ECG	Electrocardiography
eGFR	Estimated Glomerular Filtration Rate
ELISA	Enzyme-Linked Immunosorbent Assay
HDL	High-Density Lipoprotein
HU	Hounsfield Unit
IQR	Interquartile Range
MDCT	Multidetector Computed Tomography
METS-IR	Metabolic Score for Insulin Resistance
MIP	Maximum-Intensity Projection
MPR	Multiplanar Reconstruction
NHANES	National Health and Nutrition Examination Survey
OR	Odds Ratio
ROC	Receiver Operating Characteristic
SD	Standard Deviation
TyG	Triglyceride-Glucose Index
VIF	Variance Inflation Factor
